# Mismatch between fishway operation and timing of fish movements: a risk for cascading effects in partial migration systems

**DOI:** 10.1002/ece3.1937

**Published:** 2016-03-10

**Authors:** Casper H. A. van Leeuwen, Jon Museth, Odd T. Sandlund, Tore Qvenild, L. Asbjørn Vøllestad

**Affiliations:** ^1^ Department of BiosciencesCentre for Ecological and Evolutionary Synthesis (CEES)University of OsloPost Office Box 1066 Blindern0316OsloNorway; ^2^ Norwegian Institute for Nature Research (NINA)Fakkelgården2624LillehammerNorway; ^3^ Norwegian Institute for Nature Research (NINA)Høgskoleringen 97036TrondheimNorway; ^4^ The Environment Agency Hedmark CountyStatens husParkgata 362306HamarNorway

**Keywords:** Brown trout (*Salmo trutta*), eco‐evolutionary consequences, European grayling (*Thymallus thymallus*), fish passage functionality, life history strategy, salmonids

## Abstract

Habitat fragmentation is a growing problem worldwide. Particularly in river systems, numerous dams and weirs hamper the movement of a wide variety of species. With the aim to preserve connectivity for fish, many barriers in river systems are equipped with fishways (also called fish passages or fish ladders). However, few fishways provide full connectivity. Here we hypothesized that restricted seasonal opening times of fishways can importantly reduce their effectiveness by interfering with the timing of fish migration, for both spring‐ and autumn‐spawning species. We empirically tested our hypothesis, and discuss the possible eco‐evolutionary consequences of affected migration timing. We analyzed movements of two salmonid fishes, spring‐spawning European grayling (*Thymallus thymallus*) and autumn‐spawning brown trout (*Salmo trutta*), in Norway's two largest river systems. We compared their timing of upstream passage through four fishways collected over 28 years with the timing of fish movements in unfragmented river sections as monitored by radiotelemetry. Confirming our hypothesis, late opening of fishways delayed the migration of European grayling in spring, and early closure of fishways blocked migration for brown trout on their way to spawning locations during late autumn. We show in a theoretical framework how restricted opening times of fishways can induce shifts from migratory to resident behavior in potamodromous partial migration systems, and propose that this can induce density‐dependent effects among fish accumulating in lower regions of rivers. Hence, fragmentation may not only directly affect the migratory individuals in the population, but may also have effects that cascade downstream and alter circumstances for resident fish. Fishway functionality is inadequate if there is a mismatch between natural fish movements and fishway opening times in the same river system, with ecological and possibly evolutionary consequences for fish populations.

## Introduction

Habitat fragmentation is a major threat to species and an important topic in conservation biology (Nilsson et al. 2005; Noss et al. 2006). Particularly in river systems, fragmentation is pertinent due to their linear structure: obstacles such as dams and weirs directly cause fragmentation (e.g., Fuller et al. 2015). Barriers in rivers are therefore increasingly equipped with fishways, also known as fish passages, bypasses, or fish ladders (hereafter collectively referred to as fishways), allowing fishes to circumvent barriers. However, to what extent fishways restore connectivity in rivers remains debated. More and more studies assess the abundance and species composition of the fish that succeed in passing fishways (for an overview, see the recent review by Roscoe and Hinch 2010), but there is also increased attention for those fishes that are unable to pass. Among documented problems with fishways are that fish are unable to find their entrance, that fish fall back after upstream passage, and that only particular phenotypes are able to swim through the fishway (for recent reviews, see Bunt et al. 2012; Noonan et al. 2012; McLaughlin et al. 2013). As more than 45 000 large dams (height > 15 m) had been constructed worldwide by the end of the last century (Nilsson et al. 2005) and continue to be built, understanding the effectiveness of fish passages is both crucial and urgent for conservation.

Many migratory fish species use fishways as part of migrations over extensive distances during their life cycles. Individual fish often use different locations throughout the year for spawning, nursing, feeding, or overwintering, and movement can be important for their individual survival, growth, or fitness (e.g., Lucas and Baras 2001; Brönmark et al. 2013). Spawning is one of the most common motivators for long‐distance migration in fish, and can induce migrations within marine systems, between marine environments and freshwater streams (diadromous migrations) as well as between feeding and spawning areas within freshwater systems (potamodromous migrations, Lucas and Baras 2001; Brönmark et al. 2013). Fishways are crucial for connectivity in diadromous species, but are also needed for connectivity within freshwater systems. Here we concentrate on potamodromous migrations by studying freshwater fish passing through fishways within large river systems, where effects of river fragmentation might have more complex effects on populations than in the case of diadromous migrations.

One reason for this is that in potamodromous migration systems, fish populations are often partially migratory: some individuals will forage, overwinter, and spawn locally, while others migrate elsewhere in the river system to spawn (Chapman et al. 2012a,b; Dodson et al. 2013). The behavior of a phenotype that undertakes spawning migration is thereby likely determined by both a heritable and plastic component (Pulido 2011; Dodson et al. 2013; Brodersen et al. 2014). Whether or not an individual fish migrates may depend on its genotype as well as factors such as its internal physiological condition (Brodersen et al. 2008; Morita et al. 2014), environmental conditions (Brönmark et al. 2013), or the behavior of conspecifics (Kaitala et al. 1993). In potamodromous partial migration systems, fragmentation may therefore have different effects than in diadromous species. Understanding the migratory structure in a population is important when estimating potential effects of lost connectivity in river systems.

Among fishes affected by fragmentation, salmonids comprise an economically and ecologically important group (Fullerton et al. 2010). Many inland salmonids are iteroparous partial migrants that migrate upstream in river systems in either spring or autumn to spawn. For example, European grayling (*Thymallus thymallus* (L.)) typically move upstream in spring to deposit eggs in the gravel at the onset of summer (e.g., Linløkken 1993; Northcote 1995). On the other hand, species such as brown trout (*Salmo trutta*, L.), although inhabiting the same habitats, typically move upstream at the end of the summer to spawn at the onset of winter (e.g., Elliott 1994; Ovidio et al. 1998). Due to this difference in migration timing, these species encounter different circumstances during migration, and river fragmentation may differently affect their migrations.

In this study, we aimed to assess how anthropogenic barriers in rivers affect fish migration using the two largest rivers in Norway as study systems. While previous research has shown extensively that fishways are only partly effective during the times when they are operational, we here focus on the fact that many fishways around the world are not operational throughout the whole year. It is common practice to have fishways operational only during part of the year (this is the case in among others Australia, Canada, China, Mexico, Sweden, and the USA, see Table S1). We therefore specifically hypothesize that restricted seasonal operation times of fishways can constrain the migrations of both spring‐ and autumn‐migrating species, and discuss this hypothesis in a theoretical framework that highlights potential eco‐evolutionary consequences of restricted migration in partially migratory populations.

To address our hypothesis, we analyzed 28 years of data for two common salmonid species (European grayling and brown trout) passing through fishways around four dams in a 236‐km section of the Glomma river system in Norway. We compared the timing of fishway operation to (1) the timing of fish movements through these fishways and (2) the timing of natural fish movements as monitored by radiotelemetry in adjacent free‐flowing river sections. Telemetry observations were made in a free‐flowing section of the Glomma River, and a large free‐flowing section in the highly comparable Gudbrandsdalslågen River.

## Materials and Methods

### Ethics statement

Our telemetry study was approved by the National Animal Research Authority (permit numbers 2008/26156, 2009/9174, 2010/59711, 2010/56244). The fish sampling and handling procedures were an important part of these permits. Fishing permissions were obtained from the County Governors in Oppland and Hedmark, and the survey was conducted in cooperation with the landowners (the fishing right owners). In our study areas (and the whole of Norway), there is public access to land and no special permissions are needed. We used specially trained fishermen to catch fish (approved by the National Animal Research Authority) and obtained annual fishing licenses for all of them (seven persons). Brown trout and European grayling are not protected species in the study area; everybody can obtain a fishing license and fish for these species.

### Study system

**Figure 1:**
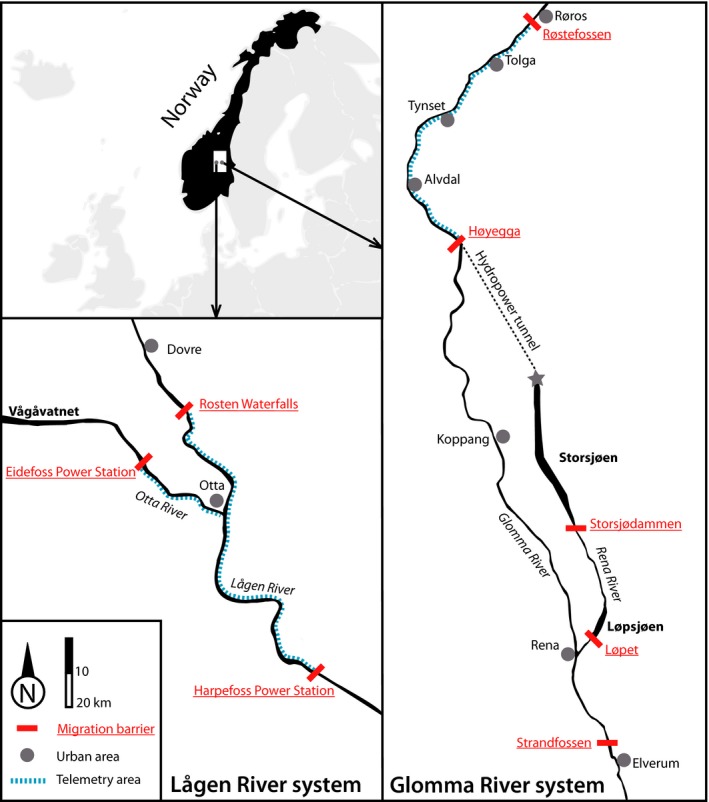
Map of the study systems Lågen River and Glomma River, Norway. Migration barriers in the rivers are indicated by (red) bars with underlined (red) names, river names are in italic, and lake names in boldface.

The rivers Glomma and Gudbrandsdalslågen (hereafter Lågen) are the two largest rivers in southeastern Norway (Fig. 1). In Glomma River, the study area covers the 236 km river section from the town of Elverum (60.832°N; 11.613°E) in the south to the dam and power plant at Røstefossen (62.507°N; 11.264°E) in the north. Downstream of Elverum salmonid densities decrease and the fish communities change significantly. The upper limit of the study site was the impassable dam at Røstefossen. The study area also included 26 km of the tributary Rena River up to Lake Storsjøen (61.394°N; 11.364°E). Within this section of the Glomma and Rena rivers, which in the natural condition was open to two‐way fish migration, there are four hydropower dams with fishways (Fig. 1, Table S2).

In the highly comparable Lågen River, the study area was the 56‐km section between the impassable downstream dam and power plant at Harpefoss (61.581°N; 9.840°E), and the upstream natural migration barrier at the waterfalls at Rosten (61.868°N; 9.411°E). This area also included 15 km of the tributary Otta up to the impassable dam and power plant at Eidefoss (61.813°N; 9.275°E). Even before dam construction, the waterfalls at Eidefoss and Harpefoss were likely barriers to upstream migration.

The Glommens and Laagens Water Association (GLB) is responsible for the monitoring and operating of hydropower dams in the two rivers, and provided detailed discharge data (1984–2012) and daily water temperatures (1996–2011) for Glomma River. For the Lågen river system, we obtained discharge data from Lalm (just above Eidefoss, 1971–2001) and Rosten Waterfalls (1971–2008) from GLB, and monitored water temperatures ourselves during 2008 and 2009 using a Hobo Pendant Temperature data logger UA‐001‐64.

### Study species

Spring‐spawning European grayling and autumn‐spawning brown trout, hereafter referred to as grayling and trout, are both rheophilic salmonids with a relatively high swimming capacity compared to many other freshwater fish species (Klemetsen et al. 2003; Clough et al. 2004). Both species are often found in complex population networks characterized by systems of partial migration (Jonsson and Jonsson 1993, 2011). Food and suitable spawning areas are often spatially and temporally heterogeneously, and habitat requirements vary between ontogenetic stages and seasons. Important driving forces for migrations are spawning, feeding, and overwintering (Klemetsen et al. 2003).

### Telemetry study

In both Glomma and Lågen river systems, a total of 180 grayling and 275 trout were captured by rod fishing throughout the study area, radio‐tagged, and subsequently released at the location of capture. The fishing was performed in the period between 2008 and 2010 in Lågen and in 2010 and 2011 in Glomma (Table 1). The telemetry data were originally collected as a part of environmental impact assessments for planned new hydropower projects in both Lågen and Glomma (see Junge et al. 2014). The purpose was to assess natural movement of both fish species in free‐flowing sections of the two river systems; hence, movement was not monitored around the dams. All fish in the Lågen and Otta rivers were tagged between Harpefoss dam, Rosten Waterfalls, and Eidefoss power station. In the Glomma system with multiple hydropower dams, all fish were tagged above Høyegga dam, and remained 40–80 km upstream of the Høyegga dam (for details, see Fig. 1). We had few radio‐tagged fish close to fishways, which limited us to assess common problems associated with fishways, such as fallbacks, difficulties of upstream passage or attraction efficiency. However, the radiotelemetry provided important knowledge about natural seasonal activity patterns in free‐flowing sections of the rivers.

**Table 1:** Details on the radiotelemetry observations of European grayling and brown trout in both river systems

Species	European grayling		Brown trout	
River	Glomma	Lågen	Glomma	Lågen
Number of individuals tracked	60	120	46	229
Years	2010–2011	2008–2010	2010–2011	2008–2010
Mean length (cm ± SD)	38.7 ± 3.3	38.6 ± 3.5	38.1 ± 5.7	40.6 ± 6.8
Length range (cm)	34–46	32–47	25–61	28–64

The same telemetry technique was used for the two fish species and in the two river systems. After capture, all fish were anaesthetized by water administered 2‐phenoxyethanol (0.7 ml·L^−1^) before placement in a cylindrical tube with well‐oxygenated water (for mounting of external transmitters) or placed with the ventral side upwards in a V‐shaped operation device (for surgical implantation of internal transmitters). The transmitter to body weight ratio never exceeded 2%. We used both internal and external radio transmitters from Advanced Telemetry Systems, Inc. Isanti, MN, USA. For 20 individuals >550 g, we used the body implant model F1830 with dimensions 12 × 54 × 12 mm, and a weight of 11 g, and for all other individuals we used either the body implant model F1580 (3 × 24 × 7 mm, 3.6 g) or the externally attached model F1970 (13 × 29 × 7 mm, 4.3 g) for logistical reasons. The external transmitters were fastened just below the dorsal fin by two stitches through the musculature (for details on the procedure, see Erkinaro et al. 1999). Internal transmitters were inserted into the abdominal cavity through a 2‐ to 3‐cm ventral incision in front of the pelvic fins, whereby the antennae were kept outside the body. Two or three Ethicon Vicryl 4‐0 absorbable sutures closed the insertion wound.

The fish were transferred to a holding tank for recovery directly after transmitter attachment (which lasted 2–4 min), and released at the capture sites approximately 30 min after they had recovered. We did not specifically assess experimentally whether or not there were adverse effects of the tagging on the fish. However, we relocated >97% of the tagged fish multiple times after initial tagging, and fast long‐distance movements of tagged fish were frequent. If tagging altered fish activity, movement would more likely decrease rather than increase. We therefore assume that any potential effects of tagging on fish behavior would not interfere with data collection for our specific question.

To locate the fish, a Challenger Receiver (model R2100; Advanced Telemetry Systems) and a three‐element folding Yagi antenna (model 12762) were operated from a car. We searched for fish year‐round in both river systems. However, our search effort was increased from mid‐March to early December in Lågen and from April to November in Glomma, with the aim to detect each fish at least once per week during this period by scanning the entire study areas.

### Fishway passage data

To improve connectivity in fish populations in Glomma River, four fishways were constructed around four hydropower dams between 1969 and 1979 (for details, see Table S2). Similar to other fishways worldwide, these fishways are not functional throughout the whole year. They typically open after the spring flood to avoid problems with woody debris in the fishways during floods, and close well before winter (see Results, and Fig. S1). The opening and closing times of the fishways are regulated by the hydropower companies and were beyond our control. However, there was variation between years in opening and closing times of fishways, enabling us to analyze possible effects of late opening or early closing. A monitoring program was started in the fishways in 1984/1985, whereby all fish entering the fishways were trapped by means of wire traps. Species, body length, and date were recorded for each fish passing through the fishways, before being released upstream of the dam. Stocked brown trout could be identified via fin clips and was excluded from this study.

### Data analyses – telemetry

**Figure 2:**
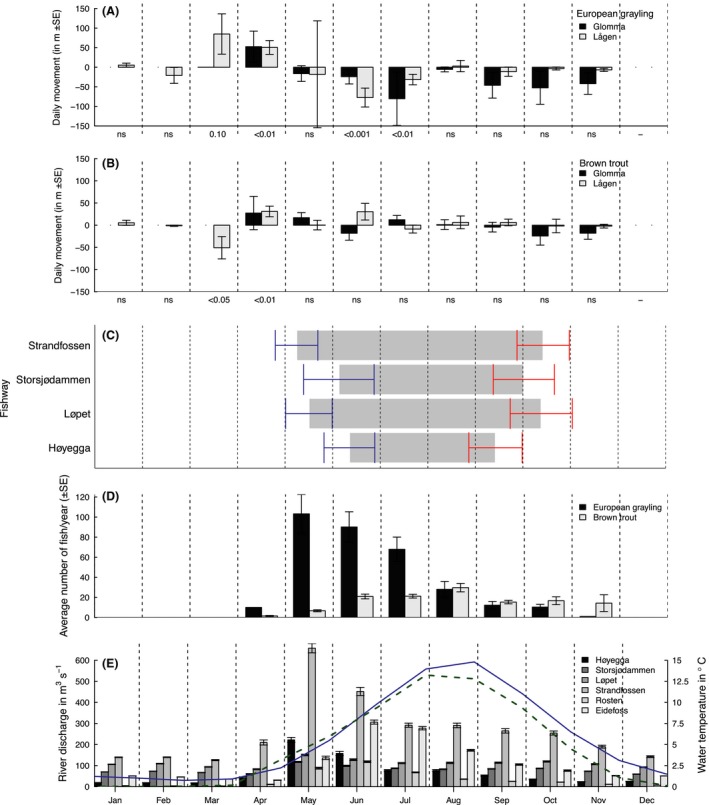
Daily movement for European grayling (A) and brown trout (B) over months of the year. Data are shown as the average movement over all tagged individuals (*n* = 180 and 275, respectively). Upstream movement is indicated as positive, downstream movement as negative. *P*‐values of one‐sample *t*‐tests testing for a significant difference in movement from 0 m are indicated for each month for the rivers combined. Error bars indicate standard errors of the mean, more statistical details in Table S3. (C) Fishway operation over months of the year. Gray bars indicate when the fishways are open. Error bars indicate SD around the mean opening and closing times during the years 1985–2011. (D) Average number of fish passing the four fishways per year indicted over months. Data are summed for the four fishways but averaged over 1985–2011. Error bars indicate SE. (E) River discharges in the two rivers. Indicated for the four different migration barriers in Glomma River and the two upstream barriers Eidefoss power plant and Rosten waterfalls in Lågen River. The solid (blue) line depicts the average river water temperature in Glomma River, and the dashed (green) line the average river water temperatures for Lågen River, both in relation to the right‐hand vertical axis.

The telemetry data were used to study the timing of fish movements in free‐flowing, unfragmented sections of the rivers. We determined whether or not species moved significantly up‐ or downstream during particular periods of the year, by classifying all movement as either upstream (positive) or downstream (negative), and averaging this per species per month. As the study systems are comparable in both biotic and abiotic characteristics and showed consistent patterns of fish movement (see Fig. 2E and Table S3 for details), the telemetry observations were pooled over 3 years to increase sample sizes and to describe behavior that is consistent over multiple years and river systems. Data were standardized to movement per day to account for differences in time intervals between consecutive detections (on average once per week). Whether or not average daily movement was significantly different from zero movement was determined per month by two‐tailed one‐sample *t*‐tests, separately per species (and by river system in Table S3). In addition, for each individual fish we calculated the length of the river section it used per month, defined as the maximum distance between the two extreme positions in the rivers where an individual was located.

### Data analyses – fishway passage

To address our hypothesis that damming would constrain migration timing of trout and grayling, we specifically tested whether late opening or early closing of fishways reduced the number of fish passing these fishways upstream. If fishways would restrict movement, we expected fewer fish to move upstream in years with more restricted opening times. We compared the number of fish that passed the fishways between years differing in opening and closing dates, using upstream passage data from the four fishways in the Glomma river system. We fitted separate models for each of the fish species. As in most of the fishways the number of passing fish has been declining over the last 28 years, and these trends differ between individual fishways and between the two species, we used a separate model for each fishway to account for different temporal autocorrelation structures. Each generalized linear model detected the unique effects in each combination of fishway and fish species (Table 2).

**Table 2:** The effects of opening and closing dates of the four fishways on the number of fish passing upstream

Species	Fishway	ARMA (*p*,*q*)^a^	Predictor variable^b^	Estimate	SE	*t*‐value	*P*‐value	Adj. *R* ^2^
Grayling	Strandfossen	None	Intercept	5.59	0.29	18.98	<0.01	−0.080
			Opening date	−0.003	0.013	−0.21	0.84	
			Closing date	0.003	0.009	0.29	0.78	
	Storsjødammen	AR (1,0)	Intercept	3.31	0.29	11.41	<0.01	−0.059
			Opening date	−0.006	0.014	−0.42	0.68	
			Closing date	0.016	0.020	0.81	0.43	
	Løpet	MA (0,1)	Intercept	5.30	4.09	1.30	0.21	−0.076
			Opening date	0.008	0.018	0.45	0.65	
			Closing date	−0.001	0.014	−0.09	0.93	
	Høyegga	AR (1,0)	Intercept	6.19	0.28	22.19	<0.01	−0.051
			Opening date	−0.009	0.013	−0.66	0.52	
			Closing date	0.006	0.017	0.35	0.73	
Trout	Strandfossen	None	Intercept	4.49	0.29	15.33	<0.01	0.20
			Opening date	0.003	0.013	0.22	0.83	
			Closing date	**0.026**	**0.009**	**2.78**	**0.010**	
	Storsjødammen	AR (1,0)	Intercept	4.80	0.13	35.70	<0.01	0.29
			Opening date	0.001	0.006	0.12	0.90	
			Closing date	**0.028**	**0.009**	**3.05**	**0.0056**	
	Løpet	AR (1,0)	Intercept	3.50	0.34	10.35	<0.01	0.32
			Opening date	0.009	0.018	0.52	0.61	
			Closing date	**0.032**	**0.012**	**2.60**	**0.016**	
	Høyegga	AR (1,0)	Intercept	4.48	0.17	26.93	<0.01	0.088
			Opening date	−0.004	0.008	−0.49	0.63	
			Closing date	0.021	0.010	2.06	0.051	

a ARMA (*p*,*q*) indicates the temporal autocorrelation structure implemented in each model: Autoregressive (AR) or by a Moving Average (MA).

b Significant predictors at the *α *= 0.05 level are indicated in boldface.

For each model, we first assessed the presence and type of temporal autocorrelation in the time series data. The potential autocorrelation and its structure was detected by testing for Autoregressive (AR), Moving Average (MA), and ARMA structures, and selecting the autocorrelation structure which resulted in the model with the lowest AIC values. These autocorrelation structures were subsequently implemented in their corresponding model (Table 2).

Each model therefore consisted of (1) the count of fish passing the fishways as quasi‐Poisson‐distributed dependent variable with log link function, which corrected for overdispersion of the data and resulted in normally distributed residuals; (2) opening Julian date and closing Julian date as centered continuous predictor variables of interest; and (3) the autocorrelation structure most suitable for the model to account for temporal changes over the years in number of passing fish. To test whether both spring‐ and autumn‐spawning species were constrained by the fishways, we specifically compared fish movement in the first 10 days after opening or the last 10 days before closing of the fishways between the two species using Welch's two‐sample *t*‐tests.

All calculations were performed using R for statistics (R‐Development‐Core‐Team 2015). Generalized linear models were computed using the package “mgcv” (Wood 2006), which allowed incorporating the temporal autocorrelation structures on non‐Gaussian distributions. Temporal autocorrelation structures were assessed using the package “forecast” (Hyndman and Khandakar 2008), and skewness of distributions was determined using the standard settings in package “e1071” (Meyer et al. 2015). Significant differences of skewness from zero were determined by comparing the skewness value to the standard error of skewness (√[6/*n*]) expected for the particular samples sizes by one‐tailed *t*‐tests (following Crawley 2013).

## Results

### Species differences in movement

Radiotelemetry data on trout and grayling indicated that both species moved considerably and directionally during early spring at low water temperatures, especially in the Lågen River (Fig. 2A and B). Significant upstream movement of grayling started as early as March in Lågen and April in Glomma (for Glomma, we have very limited data for March), and significant downstream movement occurred in June and July. Movement throughout the season ranged from 0 to as much as 29,500 m upstream during a single day. Already during early spring, grayling used considerable sections of the rivers. The average river section used by individual fish (i.e., the distance between the two extreme positions an individual fish was located) in the unfragmented sections of the rivers in March and April was 3286 ± 7095 m SD (*n* = 157), with a maximum of 61,500 m. Trout also used large sections of the river systems throughout the entire year, but did not show the sudden upstream movements as observed in grayling (see Fig. 2B and Table S3).

Counts of fish passing up the fishways confirmed the telemetry observations of movement for grayling early in the season. Many individuals moved up the fishways in May directly after opening (Fig. 2C and D). Some grayling still ascended the fishways later during the summer, but movement declined over the season (Fig. 2D). Fishway passage data indicated that the peak of grayling migration occurred in May, during the peak river discharge (Fig. 2E). A few individual fish passed the fishways already in April because the Strandfossen fishway opened before the 1^st^ of May in some years. Trout passed the fishways mainly later during the season, with most individuals passing in August (Fig. 2D).

### Effects of damming on fish movement

The fishways in our study system were only operational between May and October (Figs. 2C, S1). Over 28 years of fishway operation, the earliest opening date recorded in the Glomma river system was 17 April, but in some years some fishways opened as late as 22 July. The earliest closing date was 17 August, and the latest 13 November (Fig. S1). The fishways were on average open for 128 days ± 33 SD during 1 year (35% of the time).

The limited opening of the fishways affected fish movement, as in particular many grayling passed the fishways directly after opening. The distributions of number of passing fish were significantly skewed toward direct upstream passage after fishway opening in three of the four fishways for both species (Fig. S2). The number of fish passing the fishways during the first 10 days after opening was significantly higher for grayling than for trout. For grayling, 26.3% of all fish that passed the fishways during the season were recorded during the first 10 days after opening, while for trout the corresponding percentage was 4.5% (Welch's two‐sample *t*‐test over *n* = 27 years: *t *=* *−5.4, df = 27.4, *P *<* *0.001). In contrast, the percentage of trout passing during the 10 days before fishway closing in autumn (10.0%) was significantly higher than the corresponding percentage of grayling (2.6%, Welch's two‐sample *t*‐test over *n* = 27 years: *t *=* *4.8, df = 39.7, *P *<* *0.001). For trout, but not for grayling, the number of upstream passing fish was higher during years in which the fishways were open longer (Table 2).

## Discussion

Restricted operation times of fishways in river systems can affect migration of both spring‐ and autumn‐spawning fish species. For spring‐migrating European grayling, the four studied fishways opened on average two months after the onset of their upstream migration. This likely delayed their spawning migration, as demonstrated by a large number of individuals passing immediately after the fishways became operational. Delayed upstream migration occurred during periods of high river discharge and flooding, which implies additional costs to individuals with a migratory life history strategy. For autumn‐spawning brown trout, the number of fish that passed the fishways was lower in years with early closure of the fishways in autumn. This indicates that not all individuals could complete their intended natural migrations to their preferred spawning locations and that late migrants involuntarily had to spawn further down in the river system.

Fishways are known to only partly restore the upstream connectivity in many river systems even if they are operational, for example due to difficulties of fish to locate the entrance of the fishway, problems with swimming upstream, and fallbacks (for recent reviews, see Bunt et al. 2012; Noonan et al. 2012; McLaughlin et al. 2013). Our study highlights another potential limitation that likely applies to more fishways worldwide (see Table S1 for examples): not all fishways are continuously operational throughout the migratory season of their target species due to problems with low or high water temperatures, discharges, or maintenance. Detailed knowledge on the migration timing of the target species in river systems is needed to verify that temporary closure of fishways is indeed appropriate. Fishways should be operational throughout the entire period of the year that fish need connectivity to avoid delays, alterations, or loss of natural migration patterns in both spring‐ and autumn‐spawning fish species.

### Effects on migration timing

Spring‐migrating grayling already moved upstream in unfragmented river sections as early as March and April, and used large river sections early in the season when water temperatures were still very low. Late opening of fishways likely affected their migration timing, but did not affect the number of passing individuals. Fish seemed to wait below the fishways until these opened and subsequently passed with some delay: a pattern that is further confirmed by the high number of fish passing the first days of fishway operation. Late‐migrating trout that could not pass the fishways in autumn must either have spawned further downstream in the river system, or not at all.

Effects on migration timing can have multiple consequences for both spring‐ and autumn‐migrating species in freshwater systems. First of all, spawning of spring‐migrating fish is restricted to periods with suitable conditions in spring. In our study system, grayling generally only spawn during a 1‐ to 2‐week period in late May and early June (Junge et al. 2014). Fishways opening only shortly before or after this short period will delay spawning, cause relocation of spawning to lower in the river, or can result in a lost season. Delayed migrations are known to affect salmonid fitness, for example by limiting juvenile growth before the winter and thereby reducing juvenile survival (Kavanagh et al. 2010), exposing adults to unfavorable water temperature during migration (Keefer et al. 2008), or increasing adult predation risk (Kennedy et al. 2007). Upstream movement to areas close to the spawning grounds well before the start of spawning probably ensures timely spawning, and safeguards an individual's reproductive opportunity during the short spawning season.

Secondly, during upstream migration of European grayling in March and April the water discharge in Norwegian rivers is generally low. When fishways open in May, snowmelt has increased river discharge to at least threefold compared to that in March. The observed shift of migration timing forces grayling to migrate upstream during high river discharge, which may increase the energy required for swimming (Bohlin et al. 2001), and can increase the percentage of fish falling back after fishway passage (Reischel and Bjornn 2003).

A third potential consequence applies to both spring‐ and autumn‐spawning species. The ability to fine‐tune the timing of migration in relation to biotic and abiotic conditions is known for many migratory species, including fish (Otero et al. 2014). Phenological shifts can enable species persistence in stochastic environments and survival during changes over longer temporal scales, such as climate change (e.g., Jonzén et al. 2006; Hasler et al. 2012). Our study illustrates how damming can limit individuals to advance or delay their migration timing in response to environmental changes (e.g., climate change, early spring, altered flow patterns due to changed river banks). The combined effects of habitat fragmentation and climate change may therefore be greater than each individual effect on populations by itself.

### Consequences in partial migration systems

Our study shows that river fragmentation, if not mitigated effectively by fishways, may increase the migratory costs for both spring‐ and autumn‐migrating fish species. In potamodromous partial migration systems this might have long‐term consequences, which we here discuss in a theoretical framework to fuel future studies. Following the rationale of Alexander (1998), increasing migratory costs makes upstream spawning migration a relatively less rewarding life history strategy. Hence, hampered migration on a large scale can induce changes in life history strategies in populations. The propensity to migrate in partially migratory populations is thought to depend on a plastic as well as a heritable component (Pulido 2011; Brodersen et al. 2014). Therefore, in a first scenario, in which all individuals may flexibly balance any increasing costs of migration to their benefits, they can plastically adjust their strategies according to what is favorable in a particular season. In case of hampered migration, this can result in behavioral adjustments by fish changing their migratory to a more resident life history strategy. This could increase the proportion of resident individuals in the population (e.g., Bohlin et al. 2001; Brodersen et al. 2008). In a second scenario, in which migration is a purely genetically determined fixed trait, similar effects can be expected on longer temporal scales. More costly migration can lead to higher fitness rewards for locally spawning genotypes, and may similarly lead to a higher proportion of resident individuals.

In both scenarios, there is an anticipated increase of resident phenotypes in potamodromous migration systems. Whether due to direct plastic responses or evolutionary processes on longer timescales, an increase of resident fish may cause density‐dependent effects in downstream areas in rivers where both resident and previously migratory individuals accumulate (e.g., in spawning areas just below dams). Because density‐dependent effects can be strong in salmonids, notably during early life stages (Elliott 1994; Vøllestad et al. 2002), river fragmentation could have cascading effects by indirectly affecting the circumstances of resident individuals far downstream of barriers.

Although the theoretical framework we present is merely aimed to fuel future research and we have no possibility to test possible ecological or evolutionary consequences in our study system, our line of thinking is supported by the often large‐scale consequences of river fragmentation for entire fish populations in rivers (e.g., Zitek et al. 2008). Future empirical evidence could be obtained, for example, by (1) analyzing data on fish densities in river systems before and after fragmentation, and notably paying attention to the situation at spawning grounds directly below barriers; (2) following migratory individuals by radiotelemetry for multiple seasons in fragmented systems; (3) monitoring effects of other environmental changes on fish densities with potentially similar consequences, such as changing predation risks for migrating individuals (Hulthen et al. 2015), water temperature alterations, or the appearance of competitive invasive species on the spawning grounds of the migratory phenotype; or (4) analyzing reproductive success of resident fish populations downstream in recently fragmented rivers. Additionally, our framework could be tested in other partial migration systems than fishes, such as in birds of which land use, temperature, or predation risks change only on the breeding grounds of migratory individuals.

### Conclusions and implications

Fishways with restricted seasonal opening times can affect the migration of both spring‐ and autumn‐spawning fish. Although fish used the fishways during the 35% of the year that these were on average operational, the passages opened too late and closed too early in the season to provide connectivity throughout the entire migratory season. We showed how this may lead to spatial restructuring of fish distributions in river systems with indirect consequences for resident individuals in partial migratory systems. In addition to the many known effects of dams on fish populations, such as alterations of river morphology, water quality, and flow regimes (Mims and Olden 2013; Fuller et al. 2015), restructuring of populations can have effects that cascade far downstream from the barriers themselves. We conclude that restricted operation of fishways can limit their functionality, with ecological and possibly evolutionary consequences for freshwater fish populations.

This implies first of all that prolonging fishway opening times can increase their efficiency in situations where there is a mismatch of their opening times with the migratory timing of the target fish species. Earlier opening and later closing dates seem feasible based on the large interannual variation in current opening durations. A thorough understanding of fish movements and migratory structures of populations in river systems and improved fishway management can therefore partially increase connectivity. However, flooding, ice, or debris also often hampers operation of fishways. Therefore, not only management should be improved, but also future designs of new fishways and redesigns of existing fishways. Fishway construction should aim for full functionality throughout periods of active fish movement.

A second implication of our study is that river fragmentation can reduce the flexibility of populations to respond to environmental changes, such as climate change and reduced water quality. Populations in unfragmented landscapes can respond to environmental changes by phenological shifts, and will have higher population diversity if part of the population migrates and another part is resident. Fragmentation‐induced decreases in population diversity and/or the possibility for phenological shifts will also impair the robustness of populations when responding to environmental changes.

## Conflict of Interest

None declared.

Data is available from the Dryad Digital Repository: http://dx.doi.org/10.5061/dryad.54fv5

## Supporting information


**Appendix S1**
**Table S1.** Examples of fishways around the world that have restricted seasonal opening times.
**Table S2.** Detailed information about the dams and the four fishways in Glomma.
**Figure S1.** The opening and closing dates of the 4 fishways (Storsjødammen, Løpet, Strandfossen, and Høyegga).
**Figure S2.** Number of fish passing the fishways in relation to time after the fishway opening date in spring.

**Appendix S2**
**Table S3.** Up‐ and downstream movement of European grayling and brown trout in the river systems monitored by radiotelemetry.
