# Supplementary material for: Mismatch between fishway operation and timing of fish movements: a risk for cascading effects in partial migration systems
Source: Ecol Evol. 2016 Mar 10;6(8):2414–25. doi: 10.1002/ece3.1937 (PMC4834326; doi:10.1002/ece3.1937)
Supplement: Supplementary file 1 — Appendix S1 Table S1. Examples of fishways around the world that have restricted seasonal opening times. Table S2. Detailed information about the dams and the four fishways in Glomma. Figure S1. The opening and closing dates of the 4 fishways (Storsjødammen, Løpet, Strandfossen, and Høyegga). Figure S2. Number of fish passing the fishways in relation to time after the fishway opening date in spring. [file ECE3-6-2414-s001.docx]

**Mismatch between fishway operation and timing of fish movements: a risk for cascading effects in partial migration systems**

C. H. A. van Leeuwen, J. Museth, O.T. Sandlund, T. Qvenild and L.A. Vøllestad (2016)

*Ecology and Evolution*

**Appendix S1**

**Table S1: Examples of fishways around the world with partial operation times throughout the seasons.**

| **River** | **Country** | **Period of operation** | **Reference** |
| --- | --- | --- | --- |
| Many rivers in Victoria | Australia | Variable | (O’Brien *et al.* 2010) |
| Cobourg Brook / Big Carp River | Canada | Mar-Aug | (O’Connor *et al.* 2003) |
| Desjardins Canal | Canada | Mar-Oct | <https://www.rbg.ca/fishway> |
| Multiple rivers | China | Variable | (Chen *et al.* 2014) |
| San Juan River | Mexico | Apr-Oct | (Cheek 2014) |
| Glomma | Norway | May-Oct | This study |
| Karälven | Sweden | Jul-Aug; Sep-Oct | (Hagelin *et al.* 2015) |
| Columbia and Snake rivers | USA | Variable, commonly Apr-Aug | (DeHart 2015) |

Cheek, C. (2014) Public Service Company of New Mexico (PNM) Fish Passage Facility Annual Report 2013. Navajo Nation Department of Fish and Wildlife, Widow Rock, Arizona, USA.

Chen, K.-Q., Tao, J., Chang, Z.-N., Cao, X.-H. & Ge, H.-F. (2014) Difficulties and prospects of fishways in China: An overview of the construction status and operation practice since 2000. *Ecological Engineering,* **70,** 82-91.

DeHart, M. (2015) Fish Passage Center 2014 Annual Report. <http://www.fpc.org>, Portland, Orgeon, USA.

Hagelin, A., Calles, O., Greenberg, L., Piccolo, J. & Bergman, E. (2015) Spawning migration of wild and supplementary stocked landlocked Atlantic salmon (*Salmo salar*). *River Research and Applications***,** (early view).

O’Brien, T., Ryan, T., Stuart, I. & Saddlier, S. (2010) Review of fishways in Victoria 1996–2009. Arthur Rylah Institute for Environmental Research, Heidelberg, Victoria.

O’Connor, L., Pratt, T., Hallett, A., Katopodis, C., Bergstedt, R., Hayes, D. & McLaughlin, R. (2003) A Performance Evaluation of Fishways at Sea Lamprey Barriers and Controlled Modifications to Improve Fishway Performance. Great Lakes Fisheries Commision.

**Table S2: Detailed information about the dams and the four fishways in Glomma.** UTM position in decimal degrees, year of fishway construction, dam height in m, median water discharge in m^3^/s, the type of fishway and details on the construction for downstream migration.

|  | **Strandfossen** | **Løpet** | **Storsjødammen** | **Høyegga** |
| --- | --- | --- | --- | --- |
| **UTM** | 60.909°N  11.525°E | 61.154°N  11.456°E | 61.361°N  11.371°E | 62.020°N  10.821°E |
| **Year** | 1979 | 1971 | 1969 | 1971 |
| **Height (m)** | 13.5 | 19.3 | 10 | 10 |
| **Discharge in m^3^/s** | 158.7 | 104.2 | 74.7 | 11.8 |
| **Fishway type** | Natural stream | Pool and weir | Denil | Pool and weir |
| **Turbines** | Kaplan turbines | Kaplan turbines | - | - |
| **Spillway** | Bottom and surface water | Bottom water | Bottom and surface water | Bottom and surface water |

**Fig. S1: The opening and closing dates of the 4 fishways (Storjødammen, Løpet, Strandfossen and Høyegga).** Depicted as frequency diagrams over 28 years of data.

**Fig. S2: Number of fish passing the fishways in relation to time after the fishway opening date in spring.** Data is based on the years 1985-2011 and presented separately for all four fishways and species. Note that scaling on the y-axes differs between fishways. The skewness of the distributions is indicated in the graphs. A higher skewness means that more individuals passed shortly after the fishway opened. Average skewness for spring spawning European grayling (1.15) was higher than for autumn spawning brown trout (0.51).
